# Sex, Racial/Ethnic, and Regional Disparities in Pulmonary Embolism Mortality Trends in the USA, 1999–2020

**DOI:** 10.1007/s40615-024-02197-5

**Published:** 2024-10-25

**Authors:** Greta Muriel Eikermann, Christopher Tam, Annika Eyth, Can Martin Ludeke, Aline M. Grimme, Tina Ramishvili, Felix Borngaesser, Maira Rudolph, Nicole Aber, Sandra Emily Stoll, Corinne M. Kyriacou, Fran A. Ganz-Lord, Ibraheem M. Karaye

**Affiliations:** 1 Ethical Culture Fieldston School, New York, NY USA; 2 Department of Anesthesiology, Montefiore Medical Center, Albert Einstein College of Medicine, 111 East 210Th Street, Bronx, NY USA; 3 Intensive Care, Emergency Medicine, and Pain Therapy, Universitätsmedizin Oldenburg, University Clinic for Anesthesiology, Klinikum Oldenburg AöR, Oldenburg, Germany; 4 Faculty of Medicine and University Hospital Cologne, Department for Anesthesiology and Intensive Care Medicine, University of Cologne, Cologne, Germany; 5 Department of Internal Medicine, Montefiore Medical Center, Albert Einstein College of Medicine, Bronx, NY USA; 6 Department of Population Health, Hofstra University, Hempstead, NY USA

**Keywords:** Pulmonary embolism, Mortality, Sex disparity, Differences, Equity, Sex as a biological variable, Thrombosis

## Abstract

**Background:**

While the National Institutes of Health emphasize integrating sex as a biological variable into research, specific considerations of sex-related differences in pulmonary embolism (PE) mortality trends remain scarce. This study examines sex-based PE mortality trends across regional and demographic groups in the USA from 1999 to 2020.

**Methods:**

A retrospective analysis of National Center for Health Statistics mortality data from 1999 to 2020 was conducted. Using ICD-10 code I26, PE decedents were identified. Piecewise linear regression assessed sex-based temporal trends in PE mortality by age, race/ethnicity, and census region. Annual percentage changes and average annual percentage changes were derived using Weighted Bayesian Information Criteria. The 95% confidence intervals were estimated using the empirical quantile method.

**Results:**

From 1999 to 2020, a total of 179,273 individuals died in the USA due to PE, resulting in an age-adjusted mortality rate of 2.5 per 100,000 persons (95% CI, 2.5–2.5). While men and women exhibited comparable rates in recent time segments and across most subcategories, a higher mortality trend among males compared to females was observed among non-Hispanic White and Hispanic individuals and residents of the Western US census region. These results remained robust even after excluding data from 2020, accounting for the potential impact of the COVID-19 pandemic.

**Conclusions:**

Our study highlights sex-based disparities in PE mortality trends in the USA from 1999 to 2020. Despite overall stable mortality rates, higher trends among males were evident in specific demographic groups and regions. These findings emphasize the importance of targeted interventions to mitigate PE-related mortality discrepancies across diverse populations.

## Introduction

Pulmonary embolism (PE) represents a critical and potentially life-threatening condition characterized by the obstruction of pulmonary arteries by thrombi, leading to significant morbidity and mortality worldwide [1, 2]. PE is the third most common cause of vascular death, following myocardial infarction and stroke, and the leading preventable cause of death in hospitalized patients. The disease presents significant challenges to healthcare systems and underscores the importance of implementing effective prevention, diagnosis, and management strategies [2]. Central to understanding and addressing the impact of PE is recognizing potential variations in disease presentation, treatment outcomes, and mortality rates between males and females [3].

In examining PE-related symptoms and demographic characteristics, research consistently reveals distinctions between men and women. Women tend to be diagnosed with PE at a more advanced age and exhibit more severe symptoms and higher rates of comorbidities, including diabetes mellitus, morbid obesity, and heart failure [4, 5]. Common symptoms in women include dyspnea, syncope, and disturbed consciousness [6]. Conversely, men are typically younger at the time of PE diagnosis and have a history of smoking, lung disease, malignancy, and unprovoked PE, with chest pain and hemoptysis being more prevalent [6].

Regarding sex-based differences in PE-related mortality, findings are equivocal. Some studies report higher 30-day and 90-day mortality rates in women [4, 7, 8], while others indicate the opposite [9, 10]. However, a significant body of literature also highlights no statistically significant differences in short-term and long-term mortality between sexes [11–13]. A meta-analysis of 14 studies found no discernible sex disparity in mortality rates [6]. A prospective cohort study evaluating sex differences in PE outcomes demonstrated comparable 30-day mortality rates between men and women, independent of gender [12]. Similarly, other investigations have failed to identify significant sex-related discrepancies in mortality outcomes [14, 15].

While previous research has investigated sex differences in PE-attributed mortality rates [4, 6, 11], a significant gap persists in comprehending sex-specific trends in PE mortality within the USA. Evaluating mortality burden yields insight into historical (cumulative) deaths within a population, whereas analyzing temporal trends furnishes valuable information regarding the pace and direction of mortality changes over time [16]. This latter approach can offer insights into contemporary and future mortality patterns, thereby aiding in the development of timely intervention strategies [16].

The sole empirical study examining sex-based PE mortality trends was conducted in Spain, revealing comparable trends between genders [11]. However, due to potential variations in mortality trends across nations and the influence of individual, contextual, and environmental factors—including social determinants of health—it is imperative to investigate sex differences in PE mortality trends in the US population. This study aims to investigate PE mortality trends by sex in the USA, examining subcategories of race/ethnicity, age, and census region from 1999 to 2020.

## Methods

### Data Sources

Mortality records from 1999 to 2020 were obtained from the Centers for Disease Control and Prevention’s Wide-ranging Online Data for Epidemiologic Research [17]. This database integrates data from the National Center for Health Statistics’ Underlying Cause of Death files, which includes national mortality data derived from death certificates of all US residents. CDC WONDER has undergone validation in previous studies and has been utilized in numerous epidemiologic investigations [18–21].

Given that the present study utilized de-identified publicly accessible data, it was classified as nonhuman subjects research and exempt from institutional review board review.

### Data Abstraction

The International Classification of Diseases Codes, 10th Revision (ICD-10)-I26, was used to identify decedents whose underlying cause of death was recorded as pulmonary embolism (PE) on their death certificates. Sex-stratified crude and age-adjusted mortality rates, per 100,000, were abstracted by age, race/ethnicity, and census region.

### Statistical Analysis

Temporal trends in PE-related mortality rates were assessed using Joinpoint regression. This piecewise segmented regression approach initially assumes a uniform—linear—trend throughout the study period. Subsequently, an inflection point (Joinpoint) is incorporated into the model, and Weighted Bayesian Information Criterion (WBIC) is employed to determine its statistical significance. If the inclusion of the Joinpoint is deemed significant, it is retained; otherwise, it is excluded. This iterative process continues until the final selected model—a parsimonious model that provides a good weighted-least squared fit to the data—is derived with the optimal number of Joinpoints. The WBIC utilized in this analysis combines data-driven selection methods, namely the Bayesian Information Criterion and Bayesian Information Criterion 3, incorporating a weighted penalty term based on the data characteristics [22]. Annual percentage changes (APC) were estimated to quantify the rate of change in age-adjusted mortality rates (AAMR) within Joinpoint (piecewise) segments. Average annual percentage changes (AAPC) were computed to estimate the trends for the overall study period. The empirical quantile method (EmpQ) was utilized to derive 95% confidence intervals (CI) for the final selected model, which has been shown to yield more accurate confidence intervals. However, EmpQ is computationally intensive and, for other models, 95% CI was derived using the parametric method. Statistical significance was determined by the absence of zero in the 95% CI. A two-sided type I error of 5% was considered statistically significant for all analyses.

We accounted for the potential impact of the COVID-19 pandemic by excluding data from the year 2020 and replicating the statistical analysis.

All statistical analyses were conducted using the National Cancer Institute’s Joinpoint Regression Program 5.0.2 (Calverton, Maryland) and Stata 17.0 (College Station, TX).

## Results

Between 1999 and 2020, a total of 179,273 individuals died in the USA due to PE, resulting in an AAMR of 2.5 per 100,000 persons (95% CI, 2.5–2.5). Overall, males had a significantly higher mortality rate than females (AAMR = 2.5; 95% CI 2.5–2.5 versus AAMR = 2.4; 95% CI 2.4–2.4). This trend was also observed among Non-Hispanic White individuals, Non-Hispanic Black individuals, those aged 35–64, those aged 65 and older, and residents of the Southern US census region. However, for other demographic and regional groups, mortality rates were similar between males and females (Table 1).

**Table 1:** Sex differences in pulmonary embolism mortality rates in the USA by race/ethnicity, age, and census region, 1999–2020

Variable (*N* = 179,273)	AAMR (95% CI)	Male ^a^ AAMR (95% CI)	Female AAMR (95% CI)
Overall	2.5 (2.5–2.5)	2.5 (2.5–2.5)	2.4 (2.4–2.4)
Race/ethnicity^a^			
Non-Hispanic White	2.5 (2.5–2.5)	2.5 (2.5–2.5)	2.4 (2.4–2.4)
Non-Hispanic Black	4.5 (4.4–4.5)	4.7 (4.6–4.8)	4.4 (4.3–4.4)
Asian/Pacific Islander	0.5 (0.5–0.5)	0.5 (0.5–0.6)	0.5 (0.5–0.5)
Hispanic	1.0 (1.0–1.1)	1.0 (1.0–1.1)	1.0 (1.0–1.1)
Age (years)			
< 35	0.2 (0.2–0.2)	0.2 (0.2–0.2)	0.2 (0.2–0.2)
35–64	2.1 (2.1–2.1)	2.2 (2.1–2.2)	2.0 (2.0–2.1)
≥ 65	12.3 (12.2–12.4)	12.3 (12.2–12.4)	12.2 (12.1–12.3)
Census region			
Northeast	2.0 (2.0–2.1)	2.0 (2.0–2.1)	2.0 (2.0–2.1)
Midwest	2.8 (2.8–2.8)	2.8 (2.8–2.9)	2.8 (2.8–2.8)
South	3.0 (2.9–3.0)	3.0 (3.0–3.1)	2.9 (2.9–3.0)
West	1.6 (1.6–1.6)	1.6 (1.6–1.7)	1.6 (1.6–1.6)

*AAMR* age adjusted mortality rate

^a^Hispanics could be of any race; all other categories are non-Hispanic

### Sex Differences in PE Mortality Trends

Overall, PE mortality rates in the USA declined by 4.06% per year from 1999 to 2009 (95% CI − 5.71, − 3.02) and then stabilized from 2009 to 2020 (APC = 0.64; 95% CI − 0.27, 2.07).

When examining sex-based trends, males exhibited a stable mortality trend from 1999 through 2020. Among females, the mortality rate remained steady from 1999 to 2003 (APC =  − 1.32; 95% CI − 2.92, 1.71) but experienced a decline at an annual rate of 7.52% from 2003 to 2007 (95% CI − 9.85, − 5.60). Subsequently, the mortality rate stabilized again from 2007 to 2020 (APC = 0.05; 95% CI − 0.29, 0.44).

When stratified by age and sex, contemporary PE mortality trends showed an increase among both males and females aged below 34 years (APC = 5.26; 95% CI 1.14, 27.84 versus APC = 5.81; 95% CI 1.08, 22.86) and 35 to 64 years (APC = 2.22; 95% CI 1.50, 3.21 versus APC = 1.50; 95% CI 0.55, 3.05). However, among individuals aged 65 years and older, the most recent PE mortality trend remained stable for both sexes (APC = 0.12; 95% CI − 0.94, 1.77 versus APC =  − 0.97; 95% CI − 1.89, 0.01).

Upon stratification by race, ethnicity, and sex, contemporary mortality trends increased among non-Hispanic White males at an annual rate of 1.27% from 2008 to 2020 (95% CI 0.41, 2.47). However, rates remained stable among non-Hispanic White females (APC = 0.23; 95% CI − 0.48, 1.16). A similar observation was noted among Hispanic males, with rates increasing by 2.8% per year from 2010 to 2020 (95% CI 0.72, 6.29), while rates stabilized among Hispanic females from 2013 to 2020 (APC = 1.75; 95% CI − 1.64, 14.23). Among both non-Hispanic Black males and females, contemporary PE mortality trends were stable in the most recent time segment (APC = 1.00; 95% CI − 0.28, 3.64 versus APC = 0.74; 95% CI − 2.98, 3.40). Among Asian/Pacific Islander individuals, the trend was stationary among males (APC =  − 0.46; 95% CI − 2.82, 2.01) and declining among females (APC =  − 2.08; 95% CI − 3.83, − 0.24).

When stratified by US census region and sex, recent trends in PE mortality rates stabilized among both males and females in the northeastern (APC = 1.00; 95% CI − 0.02, 2.41 versus APC = 1.17; 95% CI − 0.40, 4.41) and midwestern regions (APC = 1.22; 95% CI − 3.26, 4.12 versus APC = 0.43; 95% CI − 0.48, 1.89). In the southern region, trends declined among males (APC =  − 4.98; 95% CI − 10.34, − 1.13) and stabilized among females (APC =  − 0.64; 95% CI − 1.43, 1.56). However, in the western region, both males (APC = 1.74; 95% CI 0.68, 3.14) and females (APC = 1.90; 95% CI 0.35, 4.47) exhibited an increase in mortality trends (Table 2).

**Table 2:** Annual percentage changes (APC) and average annual percentage changes (AAPC) in pulmonary embolism mortality rates by sex, USA, 1999–2020

Variable	Trend segment	Segment endpoints		APC (95% CI)	AAPC
		Lower	Upper		
Overall	1	1999	2009	− 4.06 (− 5.71, − 3.02)*	− 1.63 (− 2.05, − 1.20)*
	2	2009	2020	0.64 (− 0.27, 2.07)	
Sex					
Male	1	1999	2004	− 2.44 (− 4.79, 2.54)	− 1.21 (− 1.59, − 0.79)*
	2	2004	2007	− 8.64 (− 10.74, 2.28)	
	3	2007	2020	1.07 (− 0.07, 2.07)	
Female	1	1999	2003	− 1.32 (− 2.92, 1.71)	− 1.70 (− 1.91, − 1.45)*
	2	2003	2007	− 7.52 (− 9.85, − 5.60)*	
	3	2007	2020	0.05 (− 0.29, 0.44)	
Age (Years) & Sex					
< 34					
Male	1	1999	2007	− 7.18 (− 30.68, 0.43)	0.34 (− 2.17, 3.31)
	2	2007	2020	5.26 (1.14, 27.84)*	
Female	1	1999	2012	− 3.37 (− 10.29, − 1.07)*	0.03 (− 1.64, 1.61)
	2	2012	2020	5.81 (1.08, 22.86)*	
35–64					
Male	1	1999	2007	− 3.05 (− 5.19, − 1.66)*	0.18 (− 0.25, 0.62)
	2	2007	2020	2.22 (1.50, 3.21)*	
Female	1	1999	2007	− 4.40 (− 8.47, − 2.54)*	* − 0.79 (− 1.34, − 0.20)
	2	2007	2020	1.50 (0.55, 3.05)*	
≥ 65					
Male	1	1999	2009	− 5.13 (− 7.05, − 3.93)*	− 2.41 (− 2.90, − 1.91)*
	2	2009	2020	0.12 (− 0.94, 1.77)	
Female	1	1999	2004	− 2.59 (− 4.89, 2.31)	− 2.50 (− 2.85, − 2.05)*
	2	2004	2007	− 8.74 (− 10.73, 0.48)	
	3	2007	2020	− 0.97 (− 1.89, 0.01)	
Race/ethnicity and sex^a^					
Non-Hispanic White					
Male	1	1999	2008	− 4.40 (− 6.29, − 3.16)*	* − 1.20 (− 1.63, − 0.76)
	2	2008	2020	1.27 (0.41, 2.47)*	
Female	1	1999	2003	− 0.90 (− 3.92, 4.76)	− 1.37 (− 1.72, − 0.89)*
	2	2003	2007	− 6.85 (− 10.09, 0.75)	
	3	2007	2020	0.23 (− 0.48, 1.16)	
Non-Hispanic Black					
Male	1	1999	2009	− 4.08 (− 7.00, − 2.66)*	− 1.45 (− 2.04, − 0.84)*
	2	2009	2020	1.00 (− 0.28, 3.64)	
Female	1	1999	2003	− 2.24 (− 5.68, 5.10)	− 1.48 (− 2.17, − 0.83)*
	2	2003	2007	− 7.66 (− 11.81, 5.04)	
	3	2007	2020	0.74 (− 2.98, 3.40)	
Asian/Pacific Islander					
Male	1	1999	2020	− 0.46 (− 2.82, 2.01)	− 0.46 (− 2.82, 2.01)
Female	1	1999	2020	− 2.08 (− 3.83, − 0.24)*	− 2.08 (− 3.83, − 0.24)*
Hispanic					
Male	1	1999	2010	− 5.68 (− 8.20, − 3.97)*	− 1.73 (− 2.55, − 0.91)*
	2	2010	2020	2.80 (0.72, 6.29)*	
Female	1	1999	2013	− 3.91 (− 8.71, − 2.71)*	− 2.06 (− 3.13, − 1.10)*
	2	2013	2020	1.75 (− 1.64, 14.23)	
Census region and race/ethnicity					
Northeast					
Male	1	1999	2009	− 5.64 (− 7.12, − 4.48)*	− 2.22 (− 2.68, − 1.75)*
	2	2009	2020	1.00 (− 0.02, 2.41)	
Female	1	1999	2010	− 4.77 (− 7.24, − 3.44)*	− 1.99 (− 2.63, − 1.34)*
	2	2010	2020	1.17 (− 0.40, 4.41)	
Midwest					
Male	1	1999	2004	− 2.11 (− 5.20, 5.43)	− 1.06 (− 1.80, − 0.35)*
	2	2004	2007	− 8.75 (− 11.61, 5.90)	
	3	2007	2020	1.22 (− 3.26, 4.12)	
Female	1	1999	2008	− 4.66 (− 6.90, − 3.29)*	− 1.78 (− 2.25, − 1.28)*
	2	2008	2020	0.43 (− 0.48, 1.89)	
South					
Male	1	1999	2009	− 3.34 (− 4.43, − 2.46)*	− 1.68 (− 2.11, − 1.28)*
	2	2009	2017	1.74 (0.58, 5.36)*	
	3	2017	2020	− 4.98 (− 10.34, − 1.13)*	
Female	1	1999	2008	− 3.61 (− 7.07, − 2.40)*	− 1.92 (− 2.37, − 1.44)*
	2	2008	2020	− 0.64 (− 1.43, 1.56)	
West					
Male	1	1999	2008	− 5.60 (− 7.79, − 4.09)*	− 1.47 (− 1.99, − 0.94)*
	2	2008	2020	1.74 (0.68, 3.14)*	
Female	1	1999	2011	− 4.63 (− 5.99, − 3.63)*	− 1.89 (− 2.41, − 1.36)*
	2	2011	2020	1.90 (0.35, 4.47)*	

^a^Hispanics could be of any race; all other categories are non-Hispanic

^*^p-value < 0.05; 95% CI does not include zero

### Sensitivity Analysis

A sensitivity analysis utilizing PE mortality data from 1999 to 2019 revealed a slightly worsening mortality trend among males compared to females.

Overall, PE mortality trends from 1999 to 2019 initially declined at an annual rate of 4.39% from 1999 to 2008 (95% CI − 6.18, − 3.24) and then stabilized from 2008 to 2019 (APC = 0.24; 95% CI − 0.62, 1.56). Upon stratification by sex, male individuals exhibited an increase in mortality trend from 2008 to 2019 (APC = 0.99; 95% CI 0.15, 2.09), while female individuals showed a decline in mortality trend from 2007 to 2019 (APC =  − 1.83; 95% CI − 2.05, − 1.57).

When stratified by race/ethnicity and sex, non-Hispanic White and Hispanic males recorded an increasing mortality trend (APC = 1.32; 95% CI 0.31, 2.81 and APC = 2.77; 95% CI 0.27, 7.35, respectively), while their female counterparts exhibited a stable trend (APC = 0.19; 95% CI − 0.63, 1.18 and APC =  − 0.57; 95% CI − 2.38, 8.29). A similar observation was noted when trends were stratified by US census region and sex, with an increasing mortality trend among males in the Western region (APC = 1.73; 95% CI 0.39, 3.86) and a stabilizing trend among females from the same region (APC = 0.89; 95% CI − 1.01, 2.75). All other results remained consistent with our original analysis of trends from 1999 to 2020 (Table 3).

**Table 3:** Annual percentage changes (APC) and average annual percentage changes (AAPC) in pulmonary embolism mortality rates by sex, USA, 1999–2019

Variable	Trend segment	Segment endpoints		APC (95% CI)	AAPC
		Lower	Upper		
Overall	1	1999	2008	− 4.39 (− 6.18, − 3.24)*	− 1.87 (− 2.29, − 1.44)*
	2	2008	2019	0.24 (− 0.62, 1.56)	
Sex					
Male	1	1999	2008	− 4.62 (− 6.03, − 3.54)*	− 1.57 (− 1.99, − 1.16)*
	2	2008	2019	0.99 (0.15, 2.09)*	
Female	1	1999	2003	− 1.35 (− 2.93, 1.58)	− 1.83 (− 2.05, − 1.57)*
	2	2003	2007	− 7.40 (− 9.71, − 5.55)*	
	3	2007	2019	− 1.83 (− 2.05, − 1.57)*	
Age (years) and sex					
< 34					
Male	1	1999	2007	− 6.87 (− 27.51, − 0.27)*	− 0.12 (− 2.52, 2.58)
	2	2007	2019	4.64 (0.54, 25.20)*	
Female	1	1999	2012	− 3.36 (− 13.91, − 0.83)*	− 0.26 (− 2.21, 1.49)
	2	2012	2019	5.78 (− 0.27, 28.83)	
35–64					
Male	1	1999	2007	− 2.92 (− 5.11, − 1.56)*	− 0.01 (− 0.44, 0.43)
	2	2007	2019	1.98 (1.21, 3.10)*	
Female	1	1999	2003	− 0.81 (− 4.31, 5.38)	− 0.72 (− 1.23, − 0.15)*
	2	2003	2006	− 8.52 (− 10.75, 3.78)	
	3	2006	2019	1.20 (− 0.47, 2.43)	
≥ 65					
Male	1	1999	2009	− 5.21 (− 6.67, − 4.16)*	− 2.46 (− 2.93, − 1.99)*
	2	2009	2019	0.36 (− 0.72, 1.92)	
Female	1	1999	2004	− 2.58 (− 4.99, 2.48)	− 2.56 (− 3.01, − 2.06)*
	2	2004	2007	− 8.81 (− 10.90, 1.09)	
	3	2007	2019	− 0.92 (− 2.75, 0.34)	
Race/ethnicity and sex^a^					
Non-Hispanic White					
Male	1	1999	2008	− 4.42 (− 6.52, − 3.11)*	− 1.30 (− 1.79, − 0.83)*
	2	2008	2019	1.32 (0.31, 2.81)*	
Female	1	1999	2003	− 0.91 (− 3.88, 4.30)	− 1.47 (− 1.84, − 1.00)*
	2	2003	2007	− 6.81 (− 10.01, 0.91)	
	3	2007	2019	0.19 (− 0.63, 1.18)	
Non-Hispanic Black					
Male	1	1999	2008	− 4.39 (− 9.16, − 2.68)*	− 1.75 (− 2.40, − 1.07)*
	2	2008	2019	0.47 (− 0.80, 3.84)	
Female	1	1999	2003	− 2.32 (− 5.83, 5.98)	− 1.71 (− 2.60, − 0.97)*
	2	2003	2007	− 7.37 (− 11.53, 5.61)	
	3	2007	2019	0.45 (− 6.87, 3.81)	
Asian/Pacific Islander					
Male	1	1999	2019	− 0.46 (− 3.20, 2.31)	− 0.46 (− 3.20, 2.31)
Female	1	1999	2019	− 2.40 (− 4.07, − 0.74)*	− 2.40 (− 4.07, − 0.74)*
Hispanic					
Male	1	1999	2010	− 5.67 (− 8.57, − 3.89)*	− 1.96 (− 2.91, − 1.03)*
	2	2010	2019	2.77 (0.27, 7.35)*	
Female	1	1999	2009	− 4.79 (− 12.06, − 2.99)*	− 2.70 (− 3.67, − 1.72)*
	2	2009	2019	− 0.57 (− 2.38, 8.29)	
Census region and race/ethnicity					
Northeast					
Male	1	1999	2009	− 5.57 (− 7.47, − 4.31)*	− 2.45 (− 3.00, − 1.89)*
	2	2009	2019	0.78 (− 0.52, 2.90)	
Female	1	1999	2010	− 4.69 (− 7.22, − 3.42)*	− 2.24 (− 2.91, − 1.58)*
	2	2010	2019	0.85 (− 0.89, 4.95)	
Midwest					
Male	1	1999	2004	− 2.17 (− 4.60, 2.84)	− 1.29 (− 1.73, − 0.82)*
	2	2004	2007	− 8.36 (− 10.51, 2.63)	
	3	2007	2019	0.94 (− 0.80, 2.35)	
Female	1	1999	2008	− 4.51 (− 7.05, − 3.20)*	− 2.01 (− 2.51, − 1.51)*
	2	2008	2019	0.08 (− 0.93, 1.90)	
South					
Male	1	1999	2003	− 1.01 (− 3.97, 5.91)	− 1.60 (− 2.23, − 0.94)*
	2	2003	2007	− 6.37 (10.32, 1.71)	
	3	2007	2017	1.41 (0.62, 6.58)*	
	4	2017	2019	− 7.58 (− 14.09, − 1.04)*	
Female	1	1999	2004	− 1.91 (− 3.03, 0.75)	− 2.17 (− 2.50, − 1.84)*
	2	2004	2007	− 7.33 (− 8.88, − 4.45)*	
	3	2007	2016	0.29 (− 0.27, 2.14)	
	4	2016	2019	− 4.57 (− 8.82, − 1.97)*	
West					
Male	1	1999	2008	− 5.59 (− 8.49, − 3.88)*	− 1.64 (− 2.26, − 0.99)*
	2	2008	2019	1.73 (0.39, 3.86)*	
Female	1	1999	2001	3.34 (− 5.09, 10.42)	
	2	2001	2009	− 6.25 (− 10.50, 2.45)	
	3	2009	2019	0.89 (− 1.01, 2.75)	

^a^Hispanics could be of any race; all other categories are non-Hispanic individuals

^*^p-value < 0.05; 95% CI does not include zero

## Discussion

In this study, we investigated sex differences in PE mortality trends in the USA. Our analysis did not reveal a significant disparity between males and females overall, aligning with previous research conducted in Spain, indicating a lack of consistent sex differences in PE mortality rates [11]. However, we found a slightly increased trend in mortality among men compared to women for non-Hispanic White and Hispanic individuals and residents of the Western US census region.

One potential explanation for the lack of consistent sex differences in mortality trends could be that the incidence of mortality did not differ between the sexes, a notion supported by existing literature. For example, Keller et al. [12] conducted a prospective cohort study on 569 PE patients in Germany, finding no significant differences in adverse 30-day outcomes, including mortality, between men and women, regardless of sex. Similarly, other studies have reported no sex disparities in short-term and long-term mortality rates associated with PE [13, 14]. Research conducted in Alberta, Canada, and a review on the efficacy and safety of anticoagulants both found similar PE mortality rates by sex [15, 23]. A statistical meta-analysis on sex-based differences in the presentation and outcomes of PE revealed no significant difference in mortality between the sexes [6].

Another plausible explanation for the lack of sex differences in PE mortality trends may be advancements in the recognition, diagnosis, and management of PE across both sexes. These advancements, including computed tomography pulmonary angiography (CTPA), systemic thrombolysis, catheter-directed interventions, reperfusion therapy, and the establishment of pulmonary embolism response teams, coupled with improvements in healthcare infrastructure and access to specialized care, could have led to more equitable outcomes for both males and females. For instance, systemic thrombolysis is known to reduce the risk of PE-related mortality, especially in hemodynamically unstable patients [24]. Previous research has demonstrated that both sexes received reperfusion therapy equally, optimizing their survival and reducing adverse outcomes [12]. Additionally, evidence suggests no disparity between sexes in the utilization of thrombolytic therapy or IVC filter placement [6, 25]. Since its introduction in 1998, CTPA has replaced ventilation-perfusion scans and invasive angiography, with equitable utilization by sex observed in the US population. An analysis of the National Hospital Discharge Survey revealed no sex-based differences in the diagnosis of PE, utilization of diagnostic tests for PE, or duration of hospitalization for PE in the US population [26].

Moreover, increasing awareness of risk factors for PE, such as prolonged immobility, surgery, malignancy, and inherited thrombophilia, likely contributed to enhanced prevention efforts and early detection of pulmonary emboli in both sexes [25, 27]. Pharmacological thromboprophylaxis in high-risk patients and the utilization of compression stockings and early ambulation postoperatively may have also played a role in reducing the overall burden of PE mortality, irrespective of sex [28–31].

While the overall analysis did not reveal significant sex differences, specific subgroups showed a higher mortality trend among men compared to women. For example, among non-Hispanic White and Hispanic populations, males exhibited increasing mortality trends, while females showed stable trends. This pattern persisted even after accounting for the potential impact of the COVID-19 pandemic on PE mortality trends. Men displayed an increase in mortality trends compared to women, particularly among Non-Hispanic White and Hispanic individuals, and in the Western US region.

These findings underscore the necessity for further investigation into the underlying factors influencing the observed disparities in mortality trends between males and females. Potential explanations may include variations in risk factor profiles, treatment responses, disparities in healthcare utilization rates, or other sociodemographic factors. For example, men having a 5% higher tobacco use than women, a known risk factor for thromboembolic complications, could partially explain these differences [32]. However, several studies have investigated the disparities in sex and gender, revealing that biological factors and gender identity may have opposing effects on cardiovascular outcomes [33–35]. One study indicates that women reported an overall worse health-related quality of life than men, with poorer physical and mental functions [33]. These findings were more closely associated with gender-related factors, such as femininity score, than sex-based variables [33]. In another study by the same authors, it was demonstrated that while female biological sex reduced the risk of major adverse cardiovascular events by 50%, feminine gender identity increased the risk fivefold [34]. Differences in certain clinical variables, such as grip strength and metabolic syndrome, were predominantly attributable to biological sex, while others, including systolic blood pressure level, pulse wave velocity, body mass index, and depression, were more strongly influenced by gender [35]. Understanding these factors is essential for developing targeted interventions aimed at reducing PE mortality and improving outcomes for all individuals, irrespective of sex.

This study has several limitations. We focused solely on PE mortality trends, which represent the ultimate outcome of PE incidence, severity, and treatment effectiveness. Future investigations should examine other aspects of the PE continuum, such as incidence rates, access to acute care, and long-term functional outcomes, to gain a comprehensive understanding of sex differences in PE outcomes. Additionally, mortality data were abstracted from death certificate records of US decedents, which may introduce misclassification bias, especially regarding the race/ethnicity of decedents determined by visual inspection rather than obtained from informants or other documentation [36]. We defined PE using ICD codes, and the clarity of this definition depends on the accuracy of these codes. Potential errors may arise from ICD coding practices. For instance, while clinicians typically enter ICD-10 codes for diagnoses during patient admissions, medical coders may also assign these codes post-discharge or after death based on the clinician’s narrative, which can lead to errors if an incorrect code is selected initially or during transcription. Finally, we employed ecologic data, and our results must be interpreted at the national rather than individual level, acknowledging the limitation of ecologic fallacy [37].

## Conclusions

In this study of PE mortality trends in the US population, we found that overall sex differences were not consistent. However, significant disparities were observed in three specific subgroups: non-Hispanic White individuals, Hispanic individuals, and residents of the Western US census region, where males had a higher mortality trend than females. These findings emphasize the need to consider how demographic and regional factors intersect in influencing sex-based disparities in PE mortality. Further research is required to understand the underlying determinants of these disparities, including differences in risk factors, access to care, and treatment outcomes, to guide targeted interventions that address health equity in PE outcomes.

## Data Availability

Data for this study is publicly available through the CDC WONDER Underlying Cause of Death files: https://wonder.cdc.gov/ucd-icd10.html
